# Therapeutic Effects of Successful Angioplasty on the Aorta and Lower Limb Arteries on the Healing of Chronic Ischemic Wounds

**Published:** 2016-04-13

**Authors:** Mohammad Reza Zafarghandi, Morteza Taghavi, Pezhman Farshidmehr, Azadeh Sayarifard

**Affiliations:** 1 *Sina Hospital, Tehran University of Medical Sciences, Tehran, Iran.*; 2 *Center for Academic and Health Policy, Tehran University of Medical Sciences, Tehran, Iran. *

**Keywords:** *Wound healing*, *Angioplasty*, *Aorta*, *Lower extremity*

## Abstract

**Background:** Chronic wounds are a serious problem for the patient and can increase the socioeconomic burden on the healthcare system and community. This study aimed at investigating the effect of angioplasty on chronic ischemic wound healing.

**Methods:** This study was conducted in Sina Hospital, affiliated to Tehran University of Medical Sciences. Thirty-eight patients with chronic ischemic wounds and a suspicion of the narrowing or blockage of arteries underwent peripheral angiography. Arteries under angioplasty in different patients comprised the aorta and the iliac, superficial femoral, popliteal, and tibial arteries. The patients were evaluated in terms of wound healing in weekly and monthly visits. Wound healing was measured based on the Bates–Jensen criteria.

**Results:** The patients were followed up at a median of 4.5 months. The mean age of the patients was 61.1 ± 7.5 years. Of 38 patients, 12 (31.6%) were female. The involvement of arteries on angiography consisted of 16 (42.2%) cases of total occlusion and 22 (57.8%) cases of stenosis. Following angioplasty, the level of the narrowing of arteries and the wound score showed a significant reduction in all the patients (p value < 0.001). Wound healing was observed in 29 (76.3%) patients. Hematoma, pseudoaneurysm, and thrombosis comprised the complications. No significant differences were observed in terms of age, gender, and history of risk factors between the 2 groups of wound healing and nonhealing. The wound evaluation scores before (p value = 0.044) and after (p value < 0.001) angioplasty were lower in the wound healing group than in the nonhealing group.

**Conclusion:** Angioplasty of the aorta and lower limb arteries improved the healing of chronic ischemic wounds in our patients.

## Introduction

Chronic wounds are mostly defined as wounds persisting for more than 30 days without signs of healing. Chronic and treatment-resistant wounds are mainly applied to the occurrence of wounds lasting for at least 30 days without exhibiting healing signs.^1^ This phenomenon is deemed an important medical problem because it imposes a significant burden not only on the patient but also on health systems and thus society.^2^^, ^^3^ The occurrence of treatment-resistant wounds is considerably prevalent in different communities. In a study, the prevalence of treatment-resistant wounds in lower limbs was estimated at 1/50 of the general population. At ages above 70 years, the occurrence and prevalence of this complication was reported to be 9.8%.^4^^, ^^5^

Chronic wounds often manifest in lower limbs and are accompanied by different etiologies, including venous or arterial insufficiency, diabetes mellitus, and systemic factors such as nutritional status, immune suppression, and infection. Overall, tissue hypoxia is a common pathogen in creating chronic wounds.^6^^-^^9^ The prevalence of arterial perfusion defects has been reported significantly such that 24.6% of men and women above age 55 years have atherosclerotic arterial plaques and 4.5% face evident perfusion defects in peripheral arteries.^10^ Peripheral arterial disease (PAD) is an important cause of chronic ischemic wounds. PAD is the progressive stenosis or occlusion of lower limb arteries. Although the majority of individuals with PAD are asymptomatic, it can progress to critical limb ischemia (CLI) with persistent wounds and infections and finally amputation of the digits or an extremity.^11^^-^^13^ Persistent ischemic wounds on an extremity may be treated with a long-term use of antibiotics and hyperbaric therapy; nevertheless, these treatments are effective when the circulation of the injured extremity is sufficient.^14^ Surgical treatments along with endovascular techniques have been used for treating complicated patients for wound healing and limb salvage.^15^ Among therapeutic methods, endovascular revascularization, especially angioplasty, has gained popularity recently in the efforts aimed at establishing an optimal arterial flow.^16^ Endovascular revascularization is minimally invasive and is associated with low morbidity and mortality and decreased lengths of hospitalization. Successful revascularization can confer quicker improvement and restoration for patients with CLI.^17^^, ^^18^ Some studies have prospectively investigated the results of this method. It is generally believed that lower limb wounds along with arterial insufficiency have a poor restoration potential.^19^

To our knowledge, there have been hitherto limited studies on this topic in Iran. Accordingly, we designed the present study seeking to evaluate results obtained from angioplasty on the aorta and lower limb arteries regarding the healing of chronic ischemic wounds.

## Methods

The current study is a case series. The population under study consisted of patients with treatment-resistant wounds referring to Sina Hospital, a surgical referral center affiliated to Tehran University of Medical Sciences, Tehran, Iran, in 2014. The inclusion criteria consisted of chronic ischemic wounds (no improvement after 1 month of treatment) in lower limbs, angiographic evidence of the stenosis of arteries (> 50%), absence of infectious and inflammatory disorders, coagulopathy disorders, chronic liver disease, immune suppression and other contraindications to angioplasty, and willingness to participate in the study. The exclusion criterion was angioplasty failure. First, the preliminary characteristics of the patients were taken through face-to-face interviews at admission time. The patients underwent clinical and paraclinical examinations, including limb pulse evaluation and Doppler sonography or computed tomography (CT) angiography. Those with a suspicion of the narrowing or blockage of arteries, including absence of pulse on examination or radiological evidence on ultrasound or CT angiography, underwent peripheral artery angiography.

Angioplasty was performed on arteries with stenosis > 50%.^20^ Arteries under angioplasty in different patients comprised the aorta and the iliac, superficial femoral, popliteal, and tibial arteries. Angioplasty with balloons was performed on the popliteal and tibial arteries. For the superficial femoral artery, balloons were used for the stenosis, while for flow-limiting dissections and occlusions, stents were employed. For the aorta and the iliac artery, stents were used. 

The types of the stents used were the self-expandable smart stent (Cordis) and the self-expandable stent (EverFlex) for the superficial femoral artery and the middle part of the iliac artery, the self-expandable stent (OptiMed) for the aorta, and the balloon expandable stent (Visi-Pro) for the origin of the iliac artery. Antegrade angioplasty was performed on the popliteal, tibial, and the distal portion of the superficial femoral arteries. Retrograde angioplasty was performed on the aorta and on the iliac and the proximal portion of the superficial femoral arteries. The criterion for successful angioplasty was residual stenosis < 30%.^21^

Wound healing situation and need for amputation were evaluated in weekly and monthly visits. Wound healing evaluation was done based on the Bates-Jensen criteria.^22^ The classification of wound healing situation was based on the following scores: 13 to 20: minimal severity; 21 to 30: mild severity; 31 to 40: moderate severity; and 41 to 65: extreme severity.

Passing each stage and entering the next stage was the criterion for wound healing.

Data collection and recording was done through an information form specific to the study. The data encompassed demographic information, clinical history of the patents, history of medications, history of hospitalization, trauma history, and history of medical and diagnostic interventions.

In this study, the Helsinki Ethical Principles were observed. All the patients gave consent for participation in the study. No disturbance occurred during the diagnostic and medical stages, and no additional costs were imposed on the patients. All the participants were entitled to leave the study whenever they wished to.

For the statistical analyses, the statistical software SPSS, version 11.0, for Windows (SPSS Inc., Chicago, IL) was used. Means and standard deviations (means ± SDs) were used for the quantitative variables, and frequencies and frequency percentages were used for the qualitative variables. The *t*-test or the Mann–Whitney U test was employed for the comparison of the quantitative variables between the independent groups, and the paired *t*-test or the Wilcoxon signed-rank test was utilized for the dependent groups. The chi-square test was used for the comparison of the qualitative variables. In the statistical analyses, the significance level was considered < 0.05.

## Results

**Figure 1:**
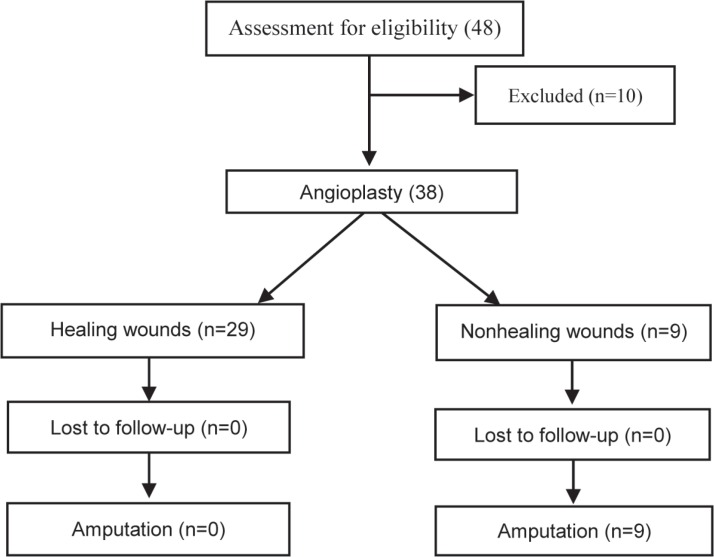
Patients’ overview

Overall, 48 patients were investigated. Ten patients were excluded due to angioplasty failures. The angioplasty failures consisted of 6 cases in the guide-wire passage in below-the-knee angioplasty, 2 cases in the guide-wire passage in the superficial femoral artery because of long occlusion, and 2 cases in the balloon passage in below-the-knee angioplasty due to heavy calcification. Finally, data of 38 patients were analyzed (Fig. 1). Twelve (31.6%) patients were female. The mean age of the patients was 61.6 ± 7.5 years, and they were between 43 and 80 years old. All the patients suffered from chronic ischemic wounds in the lower limbs.

The involvement of arteries on angiography comprised 16 (42.2%) cases of total occlusion and 22 (57.8%) cases of stenosis (Table 1). Following angioplasty, the patients were followed up for an average of 4.5 months. During this period, 29 (76.3%) patients showed wound healing and no wound healing was observed in 9 (23.7%).

**Table 1:** Patients’ angiographic characteristics*

Arteries Involved	Total Occlusion	Stenosis
Tibial	9 (56.3)	11 (50.0)
Femoral	4 (25.0)	4 (18.2)
Iliac	2 (12.5)	2 (9.1)
Aortoiliac	1 (6.2)	3 (13.6)
Popliteal	0	2 (9.1)
Total	16 (100)	22 (100)

* Data are presented as n (%).

**Figure 2:**
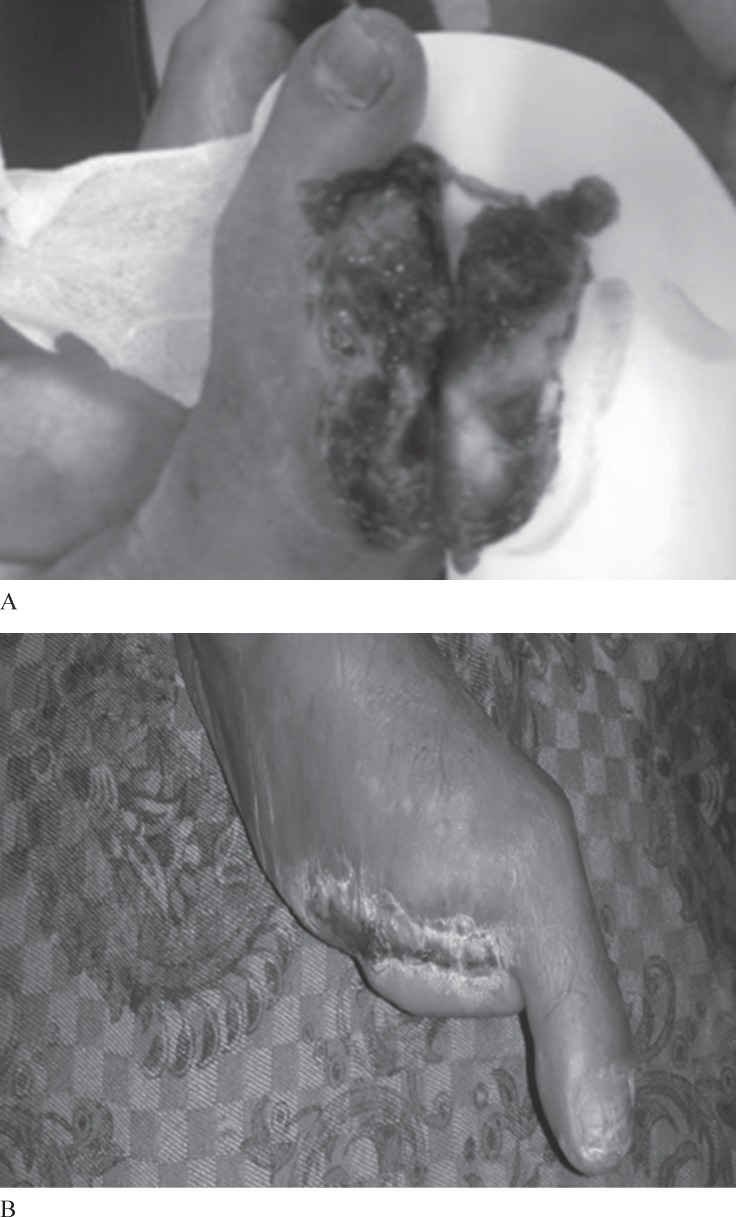
Clinical picture at admission of the patient shows a chronic wound of the right lower leg before (A) and after (B) angioplasty.

**Figure 3:**
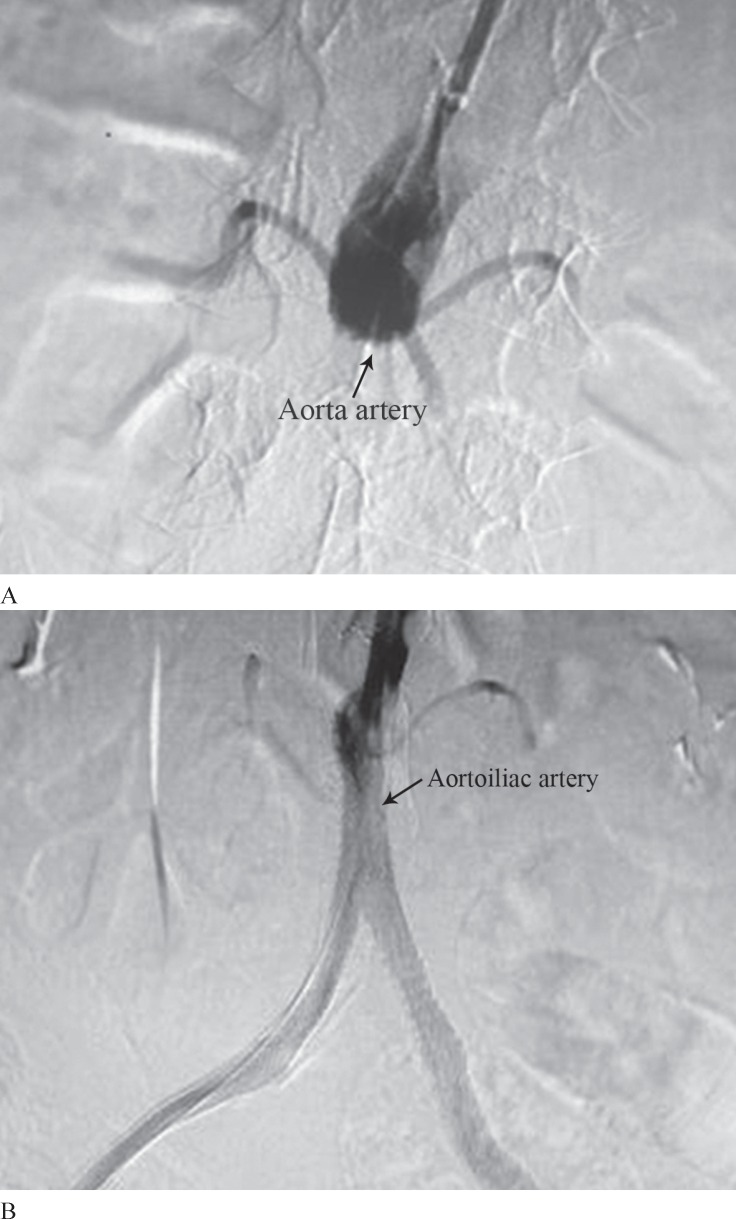
View of digital subtraction angiography (DSA) of the right aortoiliac artery before (A) and after (B) angioplasty is depicted.

Figures 2 and 3 indicate a sample of wound healing before and after angioplasty in the patients with angiographic evidence.

Following angioplasty, the percentage of artery narrowing and the wound score showed a significant reduction in all the patients (p value < 0.001). The difference in the percentage of artery narrowing before and after angioplasty was 66.8 ± 15.8 (p value < 0.001). The difference in the wound scores in the patients before and after angioplasty was 12.7 ± 13.9 (p value < 0.001).

Arrow in Figure 3-A shows total occlusion of the aorta and the arrow in Figure 3-B shows the resolution of the occlusion of the aortoiliac artery with a kissing stent.

A comparison of the characteristics of the patients between the wound healing and nonhealing groups is given in Table 2. No significant differences were seen between the 2 groups in terms of gender, age, and risk factors. Even the level of artery narrowing before angioplasty showed no significant difference between the 2 groups. Nonetheless, the wound score was significantly lower in the wound healing group before angioplasty (p value = 0.044). The mean wound score following angioplasty was 20.6 ± 5.5 in the wound healing group, while it was 54.9 ± 4.2 in the nonhealing group, which showed a significant difference (p value < 0.001).

In regard to the classification of wound healing situation, all 9 nonhealing patients were in the extreme severity class following angioplasty, while in the wound healing group, 16 (55.2%) patients were in the minimal severity class, 12 (41.4%) in the mild severity class, and 1 (3.4%) in the moderate severity class.

As regards the artery type undergoing angioplasty, in the wound healing group, 13 (48.1%) cases were on the tibial artery, 6 (22.2%) on the femoral artery, 4 (14.8%) on the popliteal artery, 2 (7.5%) on the aortic and iliac arteries, and 2 (7.4%) on the iliac artery. In the nonhealing group, 3 (50%) cases were on the tibial artery, 2 (25%) on the aortic and iliac arteries, and 2 (25%) on the iliac artery. The findings revealed no significant differences between the 2 groups (p value = 0.103). Cases of nonhealing wounds were all due to infection, and all 9 patients underwent amputation. Two (22.2%) of these patients were female.

The complications that occurred in our study consisted of hematoma, pseudoaneurysm, and thrombosis. One patient with retroperitoneal hematoma was improved by blood transfusion and modification of coagulation. One patient with large hematoma developed skin necrosis but was improved by serial debridement and granulation tissue formation. One patient with pseudoaneurysm of the common femoral artery was demonstrated to have become thrombosed on follow-up. And finally, one patient with femoral artery thrombosis underwent open surgical thrombectomy and patch angioplasty of the common femoral artery.

**Table 2:** Comparison of the characteristics of the patients between the wound healing and nonhealing groups

	Wound Healing (n=29)	Nonhealing (n=9)	P value
Age (y)	62.1±7.8	57.6±5.3	0.107
Gender (F/M)	10/19	2/7	0.489
Smoking	3 (10.3)	1 (11.1)	0.948
Hypertension	12 (41.4)	2 (22.2)	0.298
Diabetes	21 (72.4)	8 (88.9)	0.310
Hyperlipidemia	2 (6.9)	0	0.418
Artery narrowing score before angioplasty	85.2±13.3	85.6±11.3	0.938
Wound score before angioplasty	40.1±7.6	45.8±5.4	0.044

Data are presented as means±SDs or n (%).

## Discussion

Patients with chronic wounds and the narrowing or blockage of arteries in lower limbs are at high risk for amputation.^23^

Chronic wounds, especially in lower limbs, not only constitute a serious problem for the patient but also can enhance the socioeconomic burden on the healthcare system and community.^24^ It is, therefore, vitally important to have a quick and effective strategy for the treatment of chronic wounds. Inadequate perfusion due to atherosclerosis is the main factor for chronic ischemic wounds, not least in lower limbs.^25^ Gulati et al.^15^ (2015) in a review study reported that conservative and surgical treatments along with endovascular techniques created opportunities for wound healing in patients with CLI.

Endovascular techniques such as angioplasty can be employed for wound healing; however, the long-term clinical and economic consequences of these procedures remain unclear.^26^ Angioplasty allows the use of a balloon to expand a blocked or narrow artery as well as the deployment of stents and performance of atherectomy.^11^

In the present study, results obtained from angioplasty (with or without stents) on the aorta and the iliac, superficial femoral, popliteal, and tibial arteries in lower limb wound healing in 38 patients were investigated. Following angioplasty, the level of artery narrowing and the wound score showed a significant decrease in all the patients (p value < 0.001). Additionally, there was a 76% rate of wound healing success. 

The rate of wound healing in our patients is higher than that in a study by Aust et al.^27^ in Germany (2008) on patients with lower limb chronic wounds and arterial occlusion. The authors drew upon open thrombectomy or bypass surgery in addition to angioplasty along with stenting and reported successful wound healing in 21% of their patients with the absence of pulse and evidence of arterial occlusion.

The rate of wound healing reported by Crew and Thuener^28^ in the United States (1994) was better than that in the present study. The authors reported that overall 96 limbs with treatment-resistant wounds were treated using various types of endovascular techniques (Palmaz stents, angioplasty balloon, and rotational *endarterectomy*). Their findings indicated that the wounds in all the patients in the stent method, 90% in the balloon group, and 72% in the *endarterectomy* group were healed after 5 months of follow-up. Amputation cases in the *endarterectomy* group were significantly higher. The investigators underscored the stronger role of the endovascular method in the treatment of healing-resistant wounds.

Another study by Uccioli et al.^29^ investigated wound healing in diabetic patients with CLI in Italy (2010). The Texas Wound Classification was used to grade the wounds. The treatment protocol comprised rapid and extensive initial debridement, aggressive use of peripheral percutaneous angioplasty, and empirical intravenous antibiotic therapy. In contrast to our study design, the authors recruited patients with infected wounds. The study found that early diagnosis of CLI, aggressive treatment of infection, and use of percutaneous angioplasty could improve the outcome in ischemic-affected ulcers.

Another study by Yamada et al.^14^ (2013) was done on 81 cases of foot ulcer, with complications of PAD in Japan. Intravascular treatment was conducted on 69 limbs, and the initial success rate was 85.5%. Among all the patients treated, 58 had their limbs healed but 10 died. 

Research has shown that the reason for almost 9% of amputations in lower limbs is chronic wounds. In our study, unfortunately patients with nonhealing wounds underwent amputation (24%). This finding is similar to that in a review by Schamp et al.,^30^ who reported that angioplasty had a limb salvage rate of 80%. 

In the current study, no significant differences were observed between the 2 groups vis-à-vis age, gender, and history of risk factors as well as arterial narrowing on angioplasty, while the wound score (before and after angioplasty) was significantly better in the wound healing group than in the nonhealing group (p value = 0.044 and p value < 0.001).

The absence of a control group due to difficult access to an adequate sample of these patients for participation in the research project precluded us from designing a randomized controlled trial (RCT) study. We would recommend that RCTs be conducted in different medical centers to better elucidate the effect of angioplasty on chronic ischemic wound healing.

## Conclusion

Angioplasty on the aorta and lower limb arteries improved the healing of chronic ischemic wounds in our patients.

## Notes:

***This paper should be cited as:*** *Zafarghandi MR, Morteza Taghavi M, Farshidmehr P, Sayarifard A. Therapeutic Effects of Successful Angioplasty on the Aorta and Lower Limb Arteries on the Healing of Chronic Ischemic Wounds. J Teh Univ Heart Ctr 2016;11(2):79-84.*
